# Utility of Blue Light in Dermoscopy for Diagnosing Stable Lesions in Vitiligo

**DOI:** 10.5826/dpc.1101a141

**Published:** 2021-01-29

**Authors:** Balakrishnan Nirmal

**Affiliations:** 1 Department of Dermatology, Christian Medical College, Vellore, India

**Keywords:** vitiligo, amelanotic vitiligo, blue light, dermoscopy

It is important to identify the nature of the border of a vitiligo lesion to ascertain the activity of the disease. Ascertaining disease stability is an important prerequisite before subjecting the patient to surgical management. Detection of an amelanotic lesion with a sharply demarcated border (ASDB) under Wood lamp is considered stable. Unstable, active vitiligo lesions are associated with hypomelanotic appearance with poorly defined borders (HPDB) [1]. However, Wood’s lamp requires a dark room and is difficult to use in busy outpatient practice.

Loss of melanin in vitiligo is seen clearly with a 470-nm blue light source from a multispectral dermoscope (DermLite DLII, multispectral; 3Gen, San Juan Capistrano, CA). Melanin absorption is highest in the ultraviolet spectrum and decreases toward a higher wavelength. Blue light from the dermoscope has a wavelength closer to the absorption peak of melanin [2] and is useful in delineating ASDB in stable vitiligo better than white-light dermoscopy (Figure 1). HPDB seen in unstable vitiligo does not show this sharp delineation (Figure 2). Blue light increases the contrast between lesions retaining melanin and areas of melanin loss, thus is useful for differentiating stable from unstable vitiligo.

## Figures and Tables

**Figure 1:**
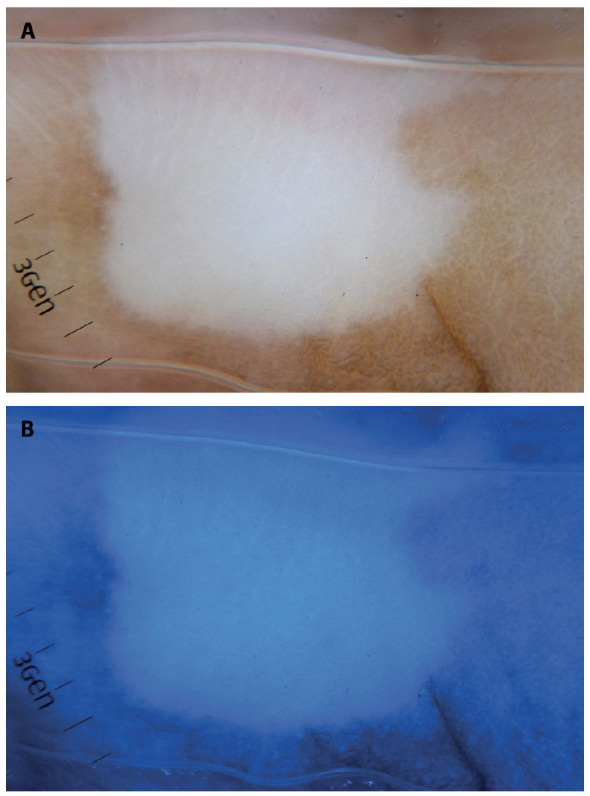
Vitiligo; stable lesion. (A) White-light and (B) blue-light dermoscopy (×10). Blue light (470 nm) delineates amelanotic vitiligo with the sharply demarcated border better than white-light dermoscopy.

**Figure 2:**
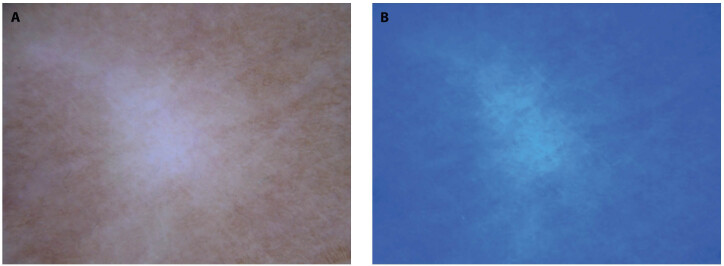
Vitiligo; unstable lesion. (A) White-light and (b) blue-light dermoscopy (×10). Blue light (470 nm) does not delineate hypomelanotic vitiligo with the poorly defined border.
